# Editorial: New Approaches in Forensic Analytical Chemistry

**DOI:** 10.3389/fchem.2020.638460

**Published:** 2021-01-12

**Authors:** Alberto Salomone, Paolo Oliveri, Grzegorz Zadora

**Affiliations:** ^1^Department of Chemistry, University of Turin, Turin, Italy; ^2^Department of Pharmacy, University of Genoa, Genoa, Italy; ^3^Forensic Chemistry Research Group, University of Silesia in Katowice, Katowice, Poland

**Keywords:** NPS, likelihood ratio, forensic science, forensic chemistry, scientific evidence

*Some place their faith in forensic science to the degree that they are under the impression that it is absolute, infallible and unassailable. In truth it is a manmade construct, dependent on manmade machinery, man-calibrated accuracy, man-led action under manmade protocols and analyzed by man – an altogether human construct* (American Academy of Forensic Sciences cited in Pyrek, 2007).

People have always strived to discover and understand the world, and the scientific quest to provide explanations fuels technological progress. This drive has fuelled forensic chemistry, where information is obtained through the examination of various evidential materials to assist the justice system piece together stories of the past. Concurrently, the validity and reliability of the information provided by forensic experts, its ability to discriminate between the standpoints of defense and prosecution, is being questioned and challenged as never before (Pyrek, 2007; Fraser and Williams, 2009). Even though analytical methods have substantially changed over time, from highly subjective assessments of information-poor data to chromatographic and spectroscopic signals, which morph into knowledge thanks to the achievements of chemometrics and statistics, forensic chemistry still is—and always will be—prone to error. The above-cited observation of the *American Academy of Forensic Sciences* is an explicit reminder of the fact that forensic science—even if increasingly enhanced with powerful computational methods—is largely a “human construct,” especially at the culmination of the examination process, which involves the interpretation and communication of findings. As a consequence, questioning the scientificity of forensics is inevitable and, thus, it is imperative to turn the focus onto the credibility of the examination process. This means that, prior to the implementation of any new forensic technique, specific steps must be taken to objectively demonstrate that the proposed methodology is suitable for its intended use (ENFSI, 2014). In other words, each of the newly developed methods has to be validated.

The role of forensic chemists is not limited solely to manufacturing valid analytical techniques and their products (physicochemical data). Many self-proclaimed forensic authors overlook the fact that these data need to be realized, as properly performed expertise also involves the interpretation and communication findings to assist fact finders (e.g., judges or prosecutors), who often lack any form of chemical knowledge or technological understanding of employed methods, in their decision making. According to the standards acknowledged among the forensic community (Zadora et al., 2014; ENFSI, 2015; Aitken et al., 2018), the communication of results should be expressed in a probabilistic manner. Any categorical conclusions are not allowed—unless the compared samples present completely different physicochemical profiles, or the results of the so-called jigsaw fitting procedure are considered—as 100% certainty can never be guaranteed. Consequently, perceiving results as categorical, and neglecting at the same time the “fuzziness of boundaries,” may lead to forensic misconduct. With that in mind, frontier research in the field of forensic chemistry should also focus on the implementation of generally accepted measures for assessing the weight of the evidence—the likelihood ratio approach—to aid the evaluation of evidence.

The articles collected in this Research Topic explore a broad range of issues that underpin the establishment of any sound analytical approach in forensic chemistry, starting from basic research, through to the development and validation of analytical tools, and the evaluation and communication of findings. When dealing with biological samples, particularly urine, extensive knowledge of the metabolic fate of substances is crucial for developing comprehensive screening procedures. Wagmann et al. studied *in vitro* approaches to investigate the metabolism of several new psychoactive substances (NPS), thus underlining the potential of zebrafish larvae as a tool for elucidating the toxicokinetics of NPS, especially when human studies are not feasible due to ethical concerns. In turn, Putz et al. performed a comprehensive *in vivo* metabolism study focused on trenbolone, a synthetic anabolic-androgenic steroid, which is misused for performance enhancement in sports. Using Hydrogen Isotope Ratio Mass Spectrometry and Liquid Chromatography/High Accuracy/High Resolution Mass Spectrometry, the authors identified new potential metabolites. A further investigation will be put in place to verify or falsify the true added value of the identified trenbolone metabolites for routine doping controls.

Given the reputation of forensic science, which has been significantly tarnished in recent years due to some infamous forensic pathologies (Trager, 2018), the challenge today is to make certain that the evidence is tested with credible analytical methods. The development of such tools is also the subject of several articles in this Research Topic. A study by Jendrzejewska addressed the authentication of popular dietary supplements containing magnesium and calcium. An X-ray structural analysis, more precisely, the comparison between diffraction lines in the recorded and reference diffraction images, provided a method for distinguishing counterfeit preparations from authentic products. Malejko et al. demonstrated that the ICP-MS method is suitable for the determination of Cd and Tl in different developmental stages of the blowfly, which—according to the authors—could be used as an alternative material for the detection of the trace element content present in the body at the time of death.

The group of NPS compounds, which are designed to mimic the activity of already existing illegal recreational drugs, receive a considerable amount of scientific interest. The continued emergence of NPS poses a number of analytical challenges for forensic laboratories. The importance of this issue is reflected in the number of NPS-directed papers submitted to this Research Topic. For example, a study authored by Calò et al. developed and validated a bioanalytical method for oral fluid analysis, using high-performance liquid chromatography coupled with mass spectrometry with minimal sample pretreatment, while Salerno et al. analyzed real “street” samples seized by law enforcement by coupling gas-chromatography to Fourier Transform Infrared Spectroscopy. Both methods proved effective for the unequivocal identification of NPS. To facilitate the work of law enforcement agencies, Bulska et al. presented a cooperative study toward the synthesis and characterization of selected NPS. The proposed non-routine analytical protocol combined X-ray diffraction with methods of chromatographic separation followed by the identification of synthesized products using mass spectrometry. Vincenti et al. reported on the successful application of molecular networking (MN) for the identification of new and unexpected fentanyl analogs within the Global Natural Product Search (GNPS), based on untargeted LC–HRMS data. The chemical structures of the compounds identified were then confirmed by NMR analysis.

Finally, Biosa et al. shed light on the interpretation of analytical data in the forensic context, with special consideration given to the likelihood ratio approach, which is now considered the most suitable framework for determining the value of forensic evidence (Zadora et al., 2014; ENFSI, 2015; Aitken et al., 2018). This particular study introduced a two-class classification strategy based on penalized logistic regression with a calculation of likelihood ratios. The method was applied to classify chronic alcohol drinkers using alcohol biomarker data. A versatile open-source and free-of-charge data processing app^1^, based on the R environment, was also presented.

## Author Contributions

AS, PO, and GZ: manuscript draft and revision. All authors contributed to the article and approved the submitted version.

## Conflict of Interest

The authors declare that the research was conducted in the absence of any commercial or financial relationships that could be construed as a potential conflict of interest.

## Footnotes

^1^https://dianagiurghita.shinyapps.io/ForensicClassification/ (accessed December 6, 2020).
